# Case Report: Anaesthetic management in a canine patient with severe atrioventricular septal defect and pulmonary hypertension undergoing non-cardiac surgery

**DOI:** 10.3389/fvets.2026.1736215

**Published:** 2026-01-29

**Authors:** Claire Nicol, Karin Kriström, Vanessa Bettembourg

**Affiliations:** Evidensia Södra Animal Hospital Kungens Kurva, Stockholm, Sweden

**Keywords:** cardiac anaesthesia, case report, congenital defect, dental procedure, dog

## Abstract

**Introduction:**

A 9-year-old, 6.8-kg intact male Petit Brabançon with a congenital partial atrioventricular septal defect (AVSD) presented for dental extractions. Pre-anaesthetic echocardiographic assessment revealed a large ostium primum atrial septal defect with bidirectional interatrial shunting, severe right atrial and ventricular enlargement, abnormal atrioventricular valves with severe tricuspid regurgitation, mild relative pulmonic stenosis, and moderate pulmonary hypertension (PH). Anaesthetic goals were to minimize increases in pulmonary vascular resistance (PVR), preserve right ventricular perfusion and cardiac output (CO), and avoid alterations in intracardiac shunt dynamics that could promote right-to-left shunting. Premedication consisted of methadone [0.2 mg/kg intramuscularly (IM)], lidocaine (1 mg/kg intravenously (IV)) and midazolam (0.3 mg/kg IV). Continuous infusions of lidocaine (30–50 μg/kg/min) and remifentanil (10–30 μg/kg/h) were used as inhalant minimum alveolar concentration (MAC)-sparing analgesic adjuncts. Anaesthesia was induced with ketamine (1 mg/kg IV) and propofol (2 mg/kg IV) and maintained with sevoflurane in oxygen and air. Controlled ventilation targeted normocapnia and the fraction of inspired oxygen (FiO₂) was titrated to maintain pulse oximeter (SpO₂) values above 95%, while avoiding unnecessary hyperoxia. A norepinephrine infusion (0.2–0.4 μg/kg/min) supported mean arterial pressure (MAP) > 65 mmHg. Regional dental nerve blocks supplemented analgesia. Fourteen teeth were extracted during a 64-min anaesthetic. Recovery was complicated by transient hypoxemia attributed to upper airway obstruction, resolving with oxygen supplementation and nebulized adrenaline.

**Outcome:**

No arrhythmias or sustained right-to-left shunting occurred. The dog was discharged the same day and returned to normal activity. Survival after the procedure was 14 months before euthanasia for acute respiratory decompensation.

**Clinical relevance:**

This report highlights practical, physiology-guided strategies to conduct anaesthesia in non-cardiac procedures in dogs with severe AVSD and PH, in hospital settings without advanced equipment such as intra-operative transesophageal echocardiography or direct CO measurements. It also serves as a reminder not to let the anaesthetic risk in patients with severe cardiac disease be the reason for refraining from quality of life improving dental procedures.

## Introduction

Atrioventricular septal defect is a rare congenital cardiac anomaly caused by incomplete fusion of the endocardial cushions during cardiac development. The defect ranges from partial to complete forms. In partial AVSD, incomplete fusion produces an ostium primum atrial septal defect and an atrioventricular valve malformation of variable severity, which may include a common atrioventricular (AV) junction with two functional orifices. In complete AVSD, the deficiency also involves the inlet portion of the ventricular septum and results in a single common AV valve overlying both atrial and ventricular septal defects (1). Large left-to-right shunts in either form, together with regurgitation from both AV valve components, increase atrial and ventricular volume, cause chamber dilation, and promote pulmonary overcirculation (2). Shunt magnitude and direction are influenced by ventricular compliance, any systemic or pulmonary outflow tract obstruction, and the balance between pulmonary and systemic vascular resistance (SVR). Developmental factors such as myocardial stiffness, pulmonary vascular maturation, and cardiopulmonary reserve may further modify shunt volume, particularly in young animals. Over time, as pulmonary vascular resistance rises, shunting may become bidirectional or reverse to right-to-left once pulmonary pressures approach systemic values (3–6).

Shunt flow also varies during the cardiac cycle. Left-to-right flow typically predominates during late ventricular systole and early diastole, and minor reversal of shunt flow may be seen in early systole and early diastole (7, 8). These cyclical fluctuations contribute to right-sided volume loading and may influence peri-anaesthetic haemodynamic stability.

Pulmonary hypertension increases right ventricle (RV) afterload, predisposing to RV hypertrophy (concentric and eccentric), dilatation, and hypoxemia, as well as arrhythmia and right-sided heart failure (9, 10). Pulmonary hypertension is defined in human medicine as a mean pulmonary artery pressure (mPAP) > 20 mmHg at rest by right heart catheterization, a threshold recently lowered from the former cutoff of ≥ 25 mmHg (11, 12). In veterinary medicine, invasive measurements are rarely performed. Instead, the American College of Veterinary Internal Medicine consensus defines PH as an increase in pulmonary arterial pressure sufficient to cause hemodynamic consequences and recommends integrating clinical signs with Doppler echocardiographic estimates, primarily tricuspid regurgitation (TR) velocity, and supportive echocardiographic indicators of right-sided pressure overload to assess PH probability and severity. A TR velocity ≥ 3.4 m/s, roughly corresponding to a systolic pulmonary arterial pressure of ~50 mmHg assuming normal right-atrial pressure, is considered a key threshold supporting a diagnosis of clinically relevant PH (13).

To the authors knowledge, there are no previous case reports specifically focusing on the anaesthetic management in canine patients with significant AVSD and PH undergoing non-cardiac surgery. We describe the peri-operative concerns and a successful anaesthetic based on pathophysiological considerations in such a patient.

## Case description

### Signalment and history

A 9-year-old, intact male Petit Brabançon (6.8 kg, body condition score: 7/9) with congenital AVSD and a common atrium diagnosed at 6 months of age, presented for dental extractions due to painful, advanced periodontal disease. Comorbidities included brachycephalic obstructive airway syndrome, chronic large-bowel disease managed with diet, and hyperadrenocorticism that remained incompletely controlled (polydipsia and polyuria) despite medical therapy. There was no history of regurgitation related to brachycephaly. The owners reported increased respiratory effort and exertional cyanosis. The current medications were pimobendan (0.3 mg/kg orally every 12 h) and trilostane (3 mg/kg orally every 24 h). Pimobendan was administered the morning of anaesthesia.

### Pre-anaesthetic evaluation

On examination, the dog was quiet, alert and responsive. The mucous membranes were pink at rest with mild cyanosis when excited. The respiratory rate was 30/min with mild effort and there were increased respiratory sounds on auscultation. A grade 2/6 systolic murmur was auscultated on the right side and the heart rate was 110/min with regular rhythm. Pulse quality was satisfactory. No arrhythmias could be seen on lead-II electrocardiogram (ECG). Oscillometric blood pressure was 140/80 mmHg (MAP 100 mmHg). Pre-operative blood work included a cephalic venous blood gas sample, packed cell volume and total solids (Table 1). Oxygen was provided via face mask during blood sampling. Full blood work including biochemistry and haematology had been done a month prior. These were within normal limits except for moderately elevated phosphate and liver enzymes.

**Table 1:** Venous blood sample results from a dog with severe atrioventricular septal defect and pulmonary hypertension before undergoing anaesthesia.

pH	7.33	7.32–7.4
pCO2	54.9	39.8–46.2 mmHg
pO2	60.3	35–45 mmHg
SO2	89.5	50–60%
HCO3	29.1	18–26 mmol/L
Base excess	3.2	–5 - –1 mmol/L
Na+	155	140–159 mmol/L
K+	3.8	3.5–5.5 mmol/L
iCa2+	1.24	1.12–1.42 mmol/L
Cl-	109	105–115 mmol/L
Glucose	6.0	3.9–6.1 mmol/L
Lactate	3.2	0–1.5 mmol/L
Packed cell volume (PCV)	42	43.3–59.3%
Total solids (TS)	7.2	6–7.5 g/dL

HCO_3_, bicarbonate; pCO_2_, partial pressure of carbon dioxide; pO_2_, partial pressure of oxygen; SO_2_, oxygen saturation.

### Echocardiographic examination

**Figure 1:**
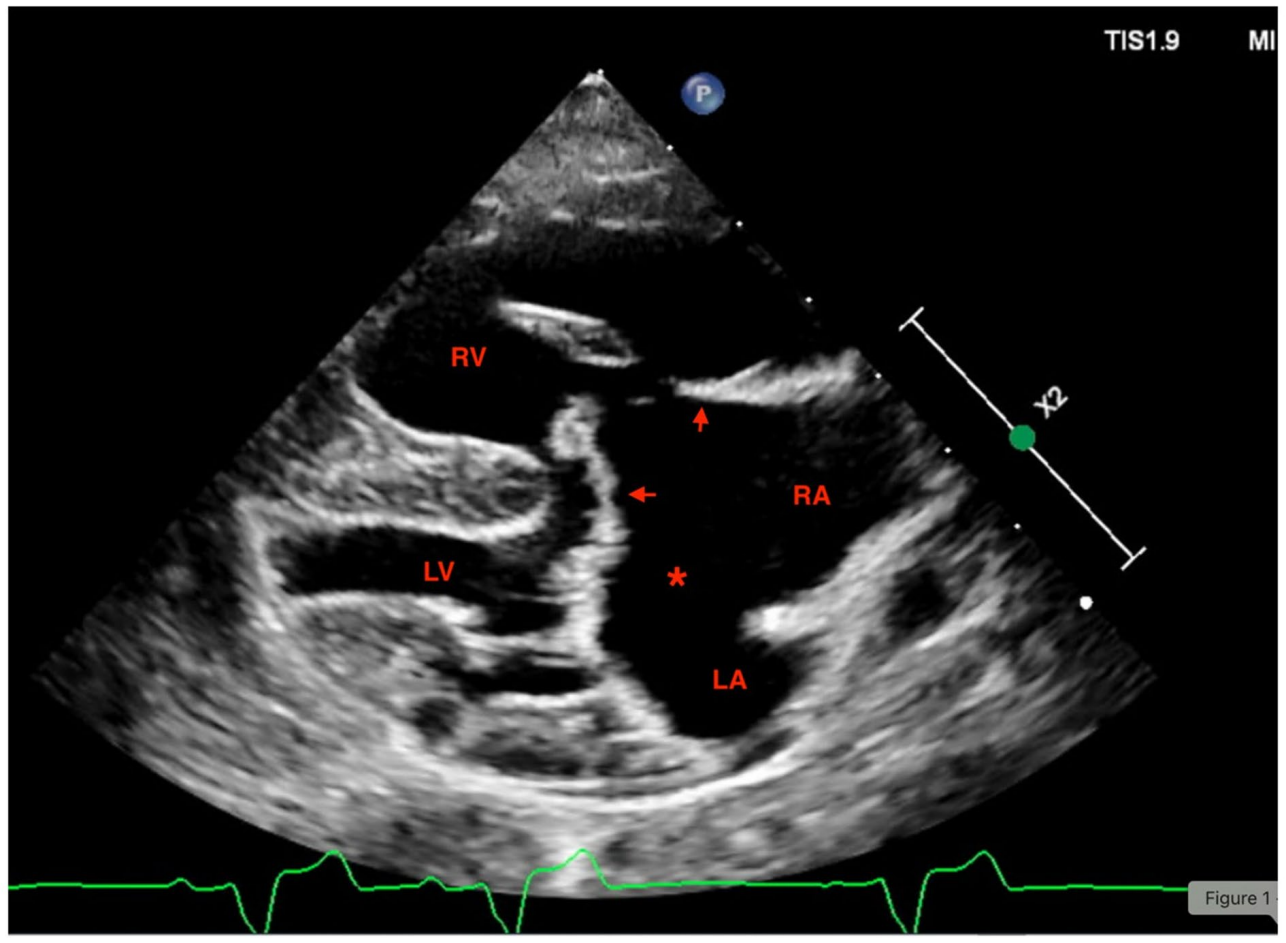
Right parasternal long axis four-chamber echocardiographic view on a dog with severe atrioventricular septal defect and pulmonary hypertension. Showing eccentric right ventricular hypertrophy, right atrial enlargement, and a large ostium primum defect (*). Arrows show the abnormal atrioventricular valves. LV, left ventricle; RV, right ventricle.

The echocardiographic examination was performed by a board-certified specialist in cardiology (KK). The dog was conscious and gently restrained in lateral recumbency. Transthoracic echocardiography was performed using an ultrasound unit equipped with 5.0–9.2 MHz phased-array transducers (EPIQ CVX; Philips Ultrasound, Bothell, WA, USA). The examination included two-dimensional (2D), M-mode, and color flow Doppler imaging and was conducted as previously described (14). A simultaneous ECG tracing was recorded during the examination, showing normal sinus rhythm. A large interatrial communication consistent with an ostium primum was visualized. A common AV valve with two separate orifices was present (Figure 1). The interventricular septum was abnormal, but a thin membrane, appearing to separate the ventricles, was present. Severe dilation of the right atrium and right ventricle was observed, with mild systolic and diastolic flattening of the interventricular septum. Right ventricular systolic function was subjectively reduced. The left ventricle was small, with a left ventricular internal diameter in diastole (LVIDd) normalized to body weight of 0.9 (15). The caudal vena cava was moderately dilated with reduced collapsibility. Bidirectional shunting was observed across the interatrial communication on agitated-saline contrast study [maximal velocity 0.8 meter per second (m/s)]. No flow was identified between the ventricles on color Doppler, excluding a ventricular septal defect. Turbulent flow with increased velocity was noted across the pulmonic valve (maximal velocity 2.2 m/s). No structural abnormalities were identified in the pulmonary artery, indicating that the pulmonic stenosis was relative. Severe tricuspid and moderate mitral regurgitation were present, with maximal velocities of 3.8 m/s (tricuspid) and 4.8 m/s (mitral). Based on the tricuspid regurgitation velocity in combination with right-sided structural abnormalities, moderate pulmonary hypertension was suspected. Thoracic radiographs revealed severe cardiomegaly, generalized interstitial pulmonary pattern, and hepatomegaly compressing the lungs.

### Anaesthetic management

Food was withheld for 6 h prior to anaesthesia. A 22 gauge intravenous cannula was placed in the left cephalic vein. He was premedicated with methadone (0.2 mg/kg IM), and midazolam (0.3 mg/kg IV). Approximately ten minutes before anaesthesia induction, a bolus of lidocaine at 1 mg/kg IV was given, followed by a continuous lidocaine infusion (30 μg/kg/min). Lidocaine and remifentanil (10 μg/kg/h) infusions were used to provide adjunct analgesia and reduce inhalant requirements and were subsequently titrated intra-operatively to 50 μg/kg/min and 30 μg/kg/h, respectively. Remifentanil was chosen due to its rapid titratability. Anaesthesia was induced with ketamine (1 mg/kg IV) and propofol (2 mg/kg IV) following 3 min of pre-oxygenation via face-mask. Because of the breed-related increased risk of regurgitation and aspiration, the head was elevated during the induction process. The trachea was intubated with a 4.5 mm ID PVC endotracheal tube with some difficulty due to an elongated soft palate.

Anaesthesia was maintained with sevoflurane (end-tidal concentration: 1.1–1.4%) delivered in an oxygen/air mixture via a circle breathing system (GE Carestation 650), with FiO₂ titrated to maintain SpO₂ > 95%. During spontaneous breathing, the dog hypoventilated (end-tidal CO_2_ (EtCO₂) 57 mmHg), likely due to opioid administration, brachycephaly, and reduced thoracic compliance associated with hepatomegaly and interstitial lung disease. Pressure-controlled ventilation was instituted to avoid hypercapnia, using inspiratory pressures of approximately 10 cmH₂O, positive end-expiratory pressure (PEEP) of 4 cmH₂O, a respiratory rate of 12–23 breaths/min and an inspiration:expiration ratio of 1:2.5 to maintain EtCO₂ within 35–45 mmHg, with care taken to limit excessive intrathoracic pressures. Just prior to initiating mechanical ventilation a transient episode of hypoxaemia (SpO2 91%) was seen which resolved with increased FiO₂ (0.6 to 0.9) and improved ventilation.

Norepinephrine (0.2–0.4 μg/kg/min) was infused to maintain MAP above 65 mmHg. Bilateral maxillary nerve blocks were performed using bupivacaine (0.3 mg/kg total; 0.2 mL per site) via a subzygomatic approach to provide regional analgesia. Multiparameter monitoring (GE B125M) included ECG, pulse oximetry, capnography, inspired/expired gas analysis, spirometry, oscillometric blood pressure, as well as Doppler blood pressure and rectal temperature. Point-of-care transthoracic Doppler ultrasound was available. However, the short episode of hypoxemia seen, resolved rapidly with initiation of mechanical ventilation and increased FiO₂, and its transient nature did not raise concern for right-to-left shunting warranting ultrasonographic confirmation. Invasive arterial blood pressure monitoring was unfortunately unavailable due to accidental dislodgement of the arterial cannula during positioning of the dog. Isotonic crystalloids (1–2 mL/kg/h) were administered, and total fluid volume including infusions did not exceed 3 mL/kg/h.

The procedure lasted 64 min. Fourteen teeth were extracted. No arrhythmia or signs of sustained right-to-left shunting occurred. Mild hypotension (MAP 55 mmHg) responded to norepinephrine titration. Early intra-operative tachycardia (peak 150/min, baseline 100/min) associated with inadequate anaesthetic depth resolved after increasing the vaporizer setting and adjusting remifentanil and lidocaine infusions. End-tidal sevoflurane remained between 1.1–1.4% with the support of MAC-sparing infusions and effective regional anaesthesia. Analgesic infusions were tapered and stopped prior to emergence. Temperature was maintained using active warming, and normothermia was preserved. Core temperature at the end of anaesthesia was 37.8 °C.

Post-operative analgesia consisted of robenacoxib (2 mg/kg subcutaneously). Pain scoring every 2 h using the Glasgow Composite Pain Scale revealed no need for rescue analgesia. Due to elongated soft palate and stenotic nares, the endotracheal tube remained in place for 15 min during recovery until the dog no longer tolerated it. Mild post-extubation hypoxemia (SpO₂ 90%) resolved with mask oxygen supplementation and nebulized adrenaline. Apart from the brief hypoxemia in recovery, no other events related to brachycephaly such as regurgitation or increased vagal tone were seen. The dog recovered uneventfully in the intensive care unit and was monitored continuously with multiparameter monitoring over 4 h without respiratory compromise or cardiovascular instability.

The owner reported normal behavior that evening and improved comfort within 48 h. The dog survived a further 14 months before euthanasia due to acute respiratory distress with cyanosis.

## Discussion

This case illustrates a pragmatic, physiology-guided anaesthetic for a non-cardiac procedure in a dog with a severe AVSD and PH. The large septal defect posed a risk of decreased CO, right sided heart failure and hypoxemia. Other anaesthetic considerations potentially affecting ventilation included obesity, hyperadrenocortisicm with hepatomegaly, and suspected interstitial lung disease. The dog was also brachycephalic which meant there was an increased risk of regurgitation, increased vagal tone, difficult intubation and hypoxemia. Our aims were to preserve RV perfusion and CO, avoiding increases in PVR, and minimising inhalant requirements using multimodal analgesia and regional anaesthesia to support SVR and CO.

During anaesthesia, a failing RV is vulnerable to decreases in coronary perfusion pressure. A recommendation from human medicine is to maintain a MAP of 65–70 mmHg to preserve RV coronary perfusion (16). However, coronary perfusion is more complex than maintaining a fixed MAP target. Coronary blood flow is determined by multiple interacting factors, including myocardial oxygen demand, intrinsic coronary autoregulation, heart rate (which modulates diastolic filling time), and autonomic nervous system tone (17). Increases in oxygen consumption, tachycardia, or sympathetic activation can significantly reduce effective RV perfusion despite an apparently adequate MAP. The systolic function of the failing RV is markedly sensitive to changes in afterload thus even minor increases in PVR can cause large decreases in stroke volume (16).

Most inhalation anaesthetics cause dose-dependent vasodilation and reductions in SVR, which can unfavourably alter shunt dynamics and compromise CO (2). To combat vasodilation, vasopressors are often needed. Norepinephrine was chosen due to its vasopressor and ionotropic properties, and its minimal chronotropic effect. This allowed preservation of coronary perfusion pressure without inducing tachycardia-associated reductions in diastolic perfusion time (18).

In patients with intracardiac shunts and PH, the balance between SVR and PVR is central to determining shunt magnitude and direction. If SVR rises disproportionately relative to PVR, blood may preferentially shunt toward the right heart, exacerbating RV volume overload and failure. Conversely, if SVR falls below PVR, right to left shunting may occur and impaired oxygen delivery may follow. Anaesthetic management therefore aimed at preventing adverse effects on shunt physiology by maintaining MAP around 65–70 mmHg and avoiding factors that would increase PVR (2, 8, 16).

Factors known to raise PVR such as hypothermia, hypercapnia, hypoxaemia, high mean airway pressure, acidosis and noxious stimulation were prevented (3, 8). Active warming was provided throughout the procedure, and normothermia was maintained. Controlled mechanical ventilation was initiated when hypercapnia was seen. Pressure-controlled ventilation was preferred over volume-controlled ventilation because it allows more predictable limitation of peak and mean airway pressures. In contrast, volume-controlled ventilation may generate injuriously high airway pressures in the presence of reduced pulmonary compliance, increasing intrathoracic pressure, impairing preload, and elevating PVR (19). Although PEEP can improve oxygenation, excessive levels may increase PVR and reduce venous return; therefore, the lowest PEEP compatible with adequate oxygenation was selected (16).

The FiO₂ was titrated to achieve a SpO₂ of ~95% to support oxygen delivery while minimising potential absorption atelectasis and decreased coronary perfusion as well as hyperoxia-induced V/Q maldistribution due to depressing hypoxic pulmonary vasoconstriction (20–22). Readiness to increase FiO₂ in case of hypoxaemia, while correcting reversible contributors (airway obstruction, secretions, positioning), was crucial.

In our effort to minimize inhalant dose and to dampen sympathetic surges from nociception, we combined systemic analgesic infusions with locoregional techniques to improve haemodynamic stability and limit PVR triggers (23, 24). To further preserve cardiovascular stability, we aimed to reduce the required dose of each induction agent by co-inducing anaesthesia with ketamine and propofol. Ketamine has been reported to increase PVR under some conditions. However, contemporary perioperative data suggest any PVR effect typically is small and may be offset by ketamine’s sympathetic support of SVR and RV contractile performance (25, 26).

In addition to afterload management, preload was considered. A hypertrophied and pressure-loaded RV is highly preload dependent, and reductions in venous return can rapidly reduce CO. Fluid therapy was administered cautiously with the aim to maintain preload without causing RV volume overload or worsening tricuspid regurgitation (16).

We avoided *α*₂-agonists and acepromazine for premedication because their haemodynamic effects (pulmonary vasoconstriction/bradycardia and systemic vasodilation, respectively) can be disadvantageous in patients with PH and intracardiac shunt physiology (18). However, in human medicine, dexmedetomidine has been used successfully and without increased complications in patients with atrial septal defect (ASD) (27). Of the case reports the authors found for cardiac corrective surgery in dogs with ASD, there were no reports of dexmedetomidine being used. The anaesthetic protocols mostly comprised of premedication with an opioid, fentanyl and lidocaine infusions throughout the procedure, induction with etomidate, propofol, alfaxalone and ketamine, and co-induction with midazolam. Both inhalant anaesthesia and total intravenous anaesthesia with propofol were described (28–32). Although total intravenous anaesthesia can limit systemic vasodilation, we elected to maintain anaesthesia with a low concentration of inhalant to facilitate rapid depth adjustments and a controlled recovery in a brachycephalic patient, meanwhile MAC-sparing infusions and vasopressor support were used to blunt inhalant-related hypotension.

Intra-operative monitoring emphasized trend recognition and rapid intervention. Invasive arterial pressure monitoring would have been ideal for continuous beat-to-beat assessment, but due to dislodgement of the arterial cannula, we relied on combined non-invasive blood pressure modalities, capnography, spirometry and gas analysis to guide therapy. Where available, intra-operative echocardiography and arterial blood gases would further refine management by quantifying shunt behavior, oxygenation and acid–base status.

The management of this case has its limitations. Invasive haemodynamic measurements and direct quantification of shunt fraction were not obtained, and protocol generalization should be made cautiously. Nevertheless, the approach anchored in maintaining MAP and SVR, preventing PVR surges, and reducing inhalant requirements with multimodal analgesia and regional techniques, is transferable to similar patients, also in settings without advanced imaging or inhaled pulmonary vasodilators.

Practical takeaways: treat dogs with significant atrial communications and PH as RV-fragile. Maintain MAP and avoid tachyarrhythmias and ischaemia, prevent PVR surges by ensuring oxygenation, normocapnia, normothermia, adequate analgesia, and cautious ventilator pressures. Usage of MAC-sparing strategies and regional blocks to limit vasodilation from inhalants can be beneficial.

## Data Availability

The original contributions presented in the study are included in the article/supplementary material, further inquiries can be directed to the corresponding author/s.
